# A dataset of deep learning performance from cross-base data encoding on MNIST and MNIST-C

**DOI:** 10.1016/j.dib.2024.111194

**Published:** 2024-12-03

**Authors:** Lawrence McKnight, Chandra Jaiswal, Issa AlHmoud, Balakrishna Gokaraju

**Affiliations:** 1601 E Market St, Greensboro, NC 27411, USA

**Keywords:** Data representation, Data abstraction, Image classification, Machine learning

## Abstract

Effective data representation in machine learning and deep learning is paramount. For an algorithm or neural network to capture patterns in data and be able to make reliable predictions, the data must appropriately describe the problem domain. Although there exists much literature on data preprocessing for machine learning and data science applications, novel data representation methods for enhancing machine learning model performance remain highly absent within the literature. This dataset is a compilation of convolutional neural network model performance trained and tested on a wide range of numerical base representations of the MNIST and MNIST-C datasets. This performance data can be further analysed by the research community to uncover trends in model performance against the numerical base of its data. This dataset can be used to produce more research of the same nature, testing cross-base data encoding on machine learning training and testing data for a wide range of real-world applications.

Specifications Table

**Table:** 

Subject	Applied Machine Learning
Specific subject area	Novel data representation and preprocessing for deep learning
Type of data	Table, Raw
Data collection	The dataset is a collection of model performance of the classification of 28 × 28 pixel images of handwritten digits 0–9 with and without corruptions (MNIST, MNIST-C).
Data source location	North Carolina A&T State University Greensboro, North Carolina, USA
Data accessibility	Repository name: cbde-data Data identification number: https://doi.org/10.5281/zenodo.13799222 Direct URL to data: https://github.com/balu-lab/cbde-data/tree/main/cbde_mnist_performance.csv The dataset is found in the above GitHub repository in file: cbde_mnist_performance.csv
Related research article	None

## 1 Value of the Data

• Practitioners can use this dataset to quickly identify statistically significant train-test base pairs, enabling them to compute directly in alternative bases without undergoing exhaustive iterative processes, thereby saving development time in their own cross-base data encoding applications.
• This dataset serves as a valuable resource for understanding the relationship between numerical base representations and model prediction accuracy, providing a foundation for further exploration of alternative bases in machine learning workflows. By using this dataset, researchers can conduct comparative studies across different domains to investigate whether numerical base encoding enhances or influences model performance, enabling new directions in model optimization.
• The dataset format can be adapted to various machine learning models and problem sets, making it a versatile tool for testing the generalizability of cross-base data encoding across different tasks and applications.

## 2 Background

Data representation is crucial in machine learning (ML) and deep learning (DL); without it, models fail to capture necessary patterns for accurate predictions. Substantial research has focused on data preprocessing techniques to optimize dataset representation for ML models. Studies show that normalization reduces model training time and enhances performance [[1], [2], [3], [4], [5]]. Dimensionality reduction has been researched extensively to minimize dataset features, improving model efficiency and enabling accurate visualization of high-dimensional data [[6], [7]]. Data augmentation has been explored to handle missing or noisy data, to generate synthetic datasets, and to model performance and training [[8], [9], [10], [11]]. Feature engineering techniques like binning, one-hot encoding, frequency encoding, and target encoding enhance problem-space representation and model accuracy [[12], [13], [14]].

Despite extensive data preprocessing research, most ML and DL studies assume base 10 data representations. However, when datasets are encoded in lower bases, distinct patterns emerge that may enhance model predictive power. This dataset aims to investigate and document the effects of cross-base data encoding on a convolutional neural network architecture for MNIST [15] and MNIST-C [16] classification, potentially uncovering new insights into model accuracy with alternative base encodings.

## 3 Data Description

A sample of the dataset is show below in Table 1. The dataset is comprised of the following columns:

• **Training base** (train): the numerical base of the training data the model was created on.
• **Testing base** (test): the numerical base of the testing data.
• **Class accuracy** (n): ten columns for each class in the MNIST data set of handwritten digits 0 through 9
• **Overall accuracy** (acc): overall performance of the model.
• **Corruption type** (corr): the corruption type of the testing data. Corruptions are either *False* if the model is testing on MNIST, or contain the following values [16]:
  ○ **identity**: No corruption is applied; the dataset remains in its original form.
  ○ **shot_noise**: Random noise following a Poisson distribution is applied, simulating conditions like low-light environments.
  ○ **impulse_noise**: Salt-and-pepper noise where random pixels are replaced with black or white values, creating isolated specks of noise.
  ○ **glass_blur**: The image is blurred in a way that simulates looking through frosted or textured glass, adding distortion.
  ○ **motion_blur**: Simulates the effect of motion by applying directional blur, similar to capturing an image with a moving object or camera.
  ○ **shear**: Applies a shearing transformation, shifting parts of the image in a specific direction, distorting the original shape.
  ○ **scale**: Scales the image up or down, simulating zoom effects and altering the appearance of the digits.
  ○ **rotate**: Rotates the image by a certain degree, testing the model's robustness to rotational variations.
  ○ **brightness**: Adjusts the brightness level, either darkening or brightening the image, simulating changes in lighting conditions.
  ○ **translate**: Shifts the image position horizontally or vertically, testing how the model handles slight positional shifts.
  ○ **stripe**: Adds horizontal or vertical stripes across the image, obscuring parts of the digits.
  ○ **fog**: Adds a foggy overlay, reducing contrast and clarity, simulating low-visibility conditions.
  ○ **spatter**: Applies a spattering effect, simulating occlusions caused by droplets or splashes on the camera lens or sensor.
  ○ **dotted_line**: Dotted lines are added across the image, introducing occlusions that make digit recognition more challenging.
  ○ **zigzag**: A zigzag pattern is overlaid onto the image, distorting the original digit shapes.
  ○ **canny_edges**: Applies the Canny edge detection algorithm, reducing the image to its edge contours, challenging models to recognize shapes without texture.

**Table 1:** Five sample rows of the dataset.

index	train	test	0	1	2	3	4	5	6	7	8	9	acc	corr
176	8	2	0.0816	0.074	0.7723	0.7129	0.0774	0.8789	0.0532	0.3589	0.0441	0.0466	0.3051	False
177	9	2	0.2265	0.3498	0.6822	0.6574	0.1273	0.7534	0.3205	0.5175	0.0554	0.0723	0.375	False
178	5	2	0.5286	0.8485	0.8236	0.9673	0.4664	0.7691	0.7881	0.8317	0.155	0.3905	0.6607	False
179	10	2	0.4224	0.5665	0.469	0.8723	0.2811	0.5785	0.4374	0.6138	0.0616	0.1596	0.4485	False
180	7	2	0.1398	0.4123	0.72	0.705	0.4308	0.7444	0.4332	0.7101	0.1961	0.2567	0.4742	False

## 4 Experimental Design, Materials and Methods

**Fig. 1:**
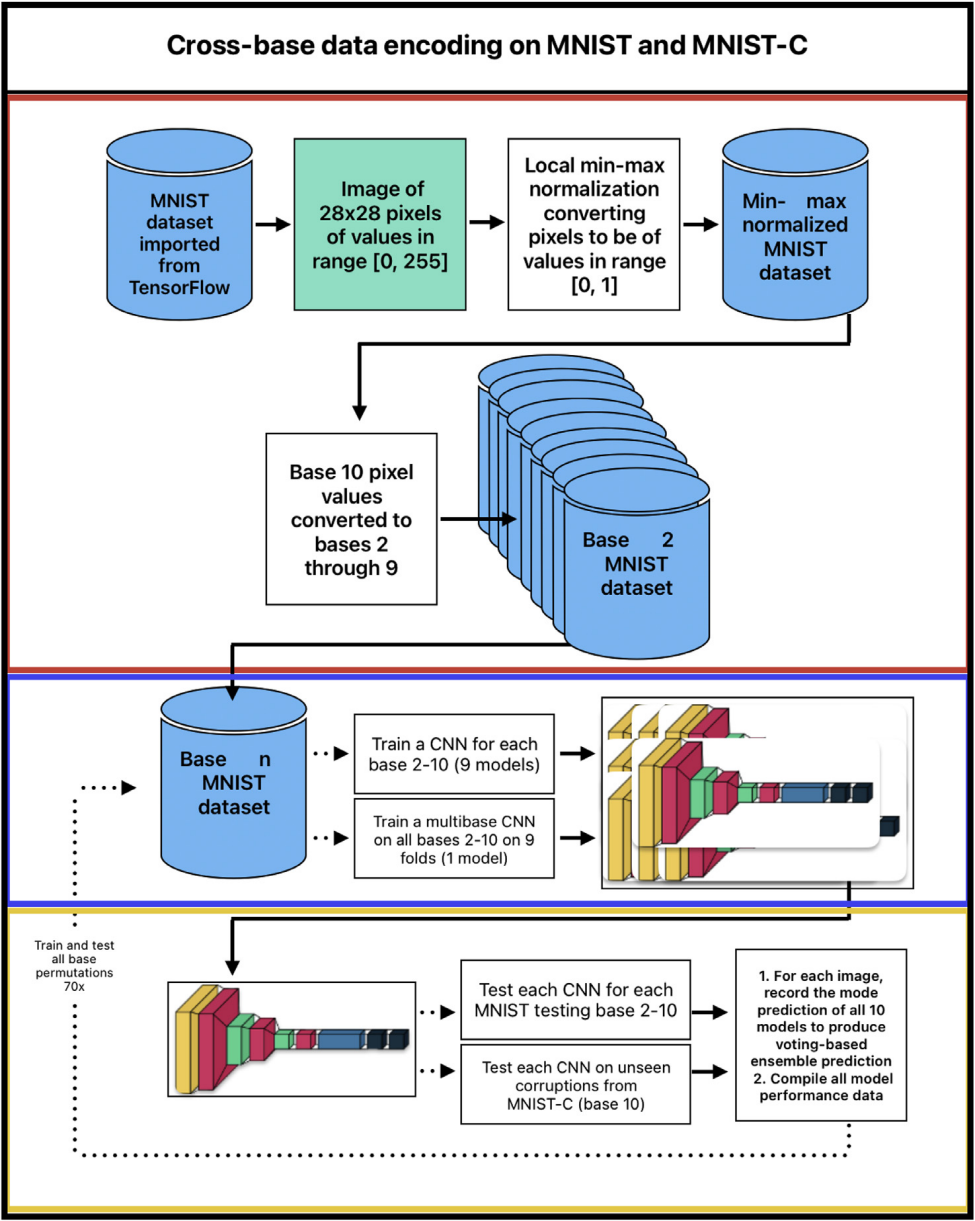
Cross-base data encoding pipeline diagram. Red: Data preparation, Blue: Model training, Yellow: Evaluation.

The dataset compiled for this paper includes class-wise accuracy data from cross-base data encoding on MNIST and MNIST-C, encompassing 70 iterations of cross-base data encoding. All models utilized a uniform convolutional neural network (CNN) architecture and were trained and tested on various encodings of the MNIST dataset. The process described in Fig. 1 involved:

1. **Model Training and Testing Data Preparation**: Pixel data from MNIST images (training and testing) were min-max normalized and converted from base 10 into bases 2 through 9, creating 8 additional datasets, and stored for repeated training and testing.
2. **Model Architecture:** The CNN was composed of three convolutional layers: the first with 32 filters and the next two with 64 filters, each using a 3 × 3 kernel and ReLU [12] activation. The first two convolutional layers are followed by max pooling layers with a 2 × 2 pool size. The result is flattened and passed through a dense layer with 64 units and ReLU activation. Finally, the model includes an output dense layer with 10 units and a softmax [13] activation function to classify the images into the ten classes of MNIST: handwritten digits 0 through 9.
3. **Model Training**: The CNN model was trained for each base representation (2 through 10), resulting in 9 trained models for 9 bases. Additionally, a multi-base model (denoted as training base 99) was trained on 9 folds, each with unique training data and a different numerical base. A voting-based ensemble (denoted as training base 999) was also created by taking the mode prediction of the nine base models for each testing record.
4. **Evaluation**: Each model was independently evaluated against all MNIST testing data bases, and class-wise accuracies were recorded. Models were also tested on unseen corrupted data (MNIST-C, base 10, no conversion), with class-wise accuracies and corruption types noted. The voting-based ensemble (999) model was compared with an ensemble of nine independent base 10 models (denoted as training base 1010) on both MNIST and MNIST-C datasets.

## Limitations

This dataset is limited to 70 iterations of class-wise performance data from CNNs trained and tested on MNIST and MNIST-C datasets encoded in bases 2 through 10. Currently, the dataset focuses on a single CNN architecture and does not include additional performance metrics such as training time or computational complexity associated with base conversions. We recognize that expanding this work to encompass multiple architectures, hyperparameter configurations, and task diversity would broaden the scope of cross-base data encoding research.

The authors are actively working on studies addressing these expanded areas, including experiments across various neural network architectures, data types, and ML/DL tasks. Nevertheless, in its present form, this dataset is designed to enable researchers to analyse the effects of CBDE on model performance and identify statistically significant alternative data representations. It empowers others to streamline CBDE into their workflows by offering a resource for evaluating data encoding effects, thus laying the groundwork for broader CBDE application in machine learning and deep learning contexts.

## Ethics Statement

The authors have adhered to the ethical requirements for publication in Data in Brief. This work does not involve human subjects, animal experiments, or data collected from social media platforms.

## Credit Author Statement

**Lawrence McKnight**: Conceptualization, Methodology, Formal Analysis, Writing – Original Draft, **Chandra Jaiswal**: Methodology, Writing – Review & Editing, **Issa AlHmoud**: Supervision, Writing – Review & Editing, **Balakrishna Gokaraju**: Supervision, Funding Acquisition.

## Data Availability

GitHubCross-Base Data Encoding MNIST Performance (Original data).
